# Neuroprotection with hypothermic reperfusion and extracorporeal cardiopulmonary resuscitation – A randomized controlled animal trial of prolonged ventricular fibrillation cardiac arrest in rats

**DOI:** 10.1177/0271678X241281485

**Published:** 2024-09-09

**Authors:** Ingrid Magnet, Alexandra-Maria Stommel, Christoph Schriefl, Matthias Mueller, Michael Poppe, Juergen Grafeneder, Christoph Testori, Andreas Janata, Andreas Schober, Daniel Grassmann, Wilhelm Behringer, Wolfgang Weihs, Michael Holzer, Sandra Hoegler, Florian Ettl

**Affiliations:** 1 Department of Emergency Medicine, 27271 Medical University of Vienna, Vienna, Austria; 2 Department of Cardiology, Klinik Floridsdorf, Vienna, Austria; 3 Emergency Medical Service Vienna, Vienna, Austria; 4 Unit of Laboratory Animal Pathology, 27260 University of Veterinary Medicine Vienna, Vienna, Austria

**Keywords:** Extracorporeal cardiopulmonary resuscitation, ventricular fibrillation cardiac arrest, neuronal damage, hypothermia, rat model

## Abstract

Extracorporeal cardiopulmonary resuscitation (ECPR) facilitates resuscitation with immediate and precise temperature control. This study aimed to determine the optimal reperfusion temperature to minimize neurological damage after ventricular fibrillation cardiac arrest (VFCA). Twenty-four rats were randomized (n = 8 per group) to normothermia (NT = 37°C), mild hypothermia (MH = 33°C) or moderate hypothermia (MOD = 27°C). The rats were subjected to 10 minutes of VFCA, before 15 minutes of ECPR at their respective target temperature. After ECPR weaning, rats in the MOD group were rapidly rewarmed to 33°C, and temperature maintained at 33°C (MH/MOD) or 37°C (NT) for 12 hours before slow rewarming to normothermia (MH/MOD). The primary outcome was 30-day survival with overall performance category (OPC) 1 or 2 (1 = normal, 2 = slight disability, 3 = severe disability, 4 = comatose, 5 = dead). Secondary outcomes included awakening rate (OPC ≤ 3) and neurological deficit score (NDS, from 0 = normal to 100 = brain dead). The survival rate did not differ between reperfusion temperatures (NT = 25%, MH = 63%, MOD = 38%, p = 0.301). MH had the lowest NDS (NT = 4[IQR 3–4], MH = 2[1–2], MOD = 5[3–5], p = 0.044) and highest awakening rate (NT = 25%, MH = 88%, MOD = 75%, p = 0.024). In conclusion, ECPR with 33°C reperfusion did not statistically significantly improve survival after VFCA when compared with 37°C or 27°C reperfusion but was neuroprotective as measured by awakening rate and neurological function.

## Introduction

Extracorporeal cardiopulmonary resuscitation (ECPR) is the use of extracorporeal membrane oxygenation (ECMO) during cardiac arrest (CA) to restore systemic perfusion and oxygenation beyond the capabilities of chest compressions and ventilation, allowing control of reperfusion conditions such as blood flow and pressure, oxygenation, and decarboxylation, reperfusate composition and temperature. ECPR has expanded the limits of cardiopulmonary resuscitation (CPR) in terms of CA pathologies and low-flow durations that can be survived with good neurological recovery.^1,2^ While the best integration of ECPR into existing treatment algorithms remains unclear,^3^ recent resuscitation guidelines have highlighted ECPR as a crucial intervention for selected patient group.^4^

Mechanisms of ischaemia-reperfusion brain injury after CA, including endothelial dysfunction, free radical formation, mitochondrial dysfunction, excitotoxicity and apoptosis, are temperature dependent in the experimental setting.^5^ Temperature control to mitigate ischaemia-reperfusion injury in comatose patients after CA has been extensively studied,^6,7^ but the evidence is conflicting, and the optimal temperature target remains a matter of debate between 32°C and fever-prevention (<37.5°C).^8,9^ None of the studies contributing to current guidelines and discussions on temperature control have included ECPR patients, who may have more severe ischaemia-reperfusion injury from prolonged low-flow times compared to patients after successful conventional CPR, and extrapolation of findings from studies in conventional CPR to the ECPR population may not be appropriate. In ECPR patients, well-controlled hypothermia achieved with the heat exchanger on the ECMO oxygenator has been associated with improved neurological outcome.^10^ Lower temperature targets, which have not been shown to improve neurological outcomes after conventional CPR,^11,12^ may be beneficial in ECPR, especially during the early phase of reperfusion.^13^ However, the optimal target temperature for ECPR patients during initial reperfusion remains unknown.

## Study objective

The aim of this randomized controlled trial was to compare the effect of three different ECPR reperfusion temperature targets after 10 minutes of ventricular fibrillation CA (VFCA) on survival, neurological and brain histological outcomes in rats. We hypothesized that immediate mild hypothermic (33°C) reperfusion after prolonged VFCA, compared to normothermic (37°C) or moderate hypothermic (27°C) reperfusion, would reduce ischaemia-reperfusion injury and improve survival with good neurological outcome.

## Material and methods

### Study design

In this randomized controlled study, rats were assigned to normothermic (NT, 37°C), mild hypothermic (MH, 33°C), or moderate hypothermic (MOD, 27°C) body temperature during ECPR reperfusion (n = 8 per group; supplemental Figure 1) using sequentially numbered, opaque sealed envelopes after successful preparation. If predefined adverse events occurred after randomization (equipment failure, air embolism during ECMO run, or operation site bleeding during decannulation)^14^ rats were excluded from the study and the and the experiment repeated on the next study day. During the study intervention, blinding was not possible due to different temperature levels. Blinding was implemented after resuscitation for neurological and histological outcome evaluation by identifying rats with nondescript tail markings and random housing, and encrypted numbering of histological samples by technical staff. The study protocol followed Austrian federal law (§ 16 Tierversuchsgesetz, TVG 2012), Directive 2010/63/EU and ARRIVE guidelines,^15^ and was approved by the Institutional Ethics Committee for Laboratory Animal Experiments of the Medical University of Vienna (GZ.: 66.009/0064-II/3b/2011).

### Animal care and handling

Adult male Sprague-Dawley rats (10–12 weeks, 450–500 g body weight [BW], division of laboratory animal science and genetics, Himberg, Austria) were housed in small groups under standard conditions (room temperature, 12-hour light/dark cycle, unrestricted access to food and water). Following resuscitation, rats received supportive care including subcutaneous buprenorphine for distress/pain, subcutaneous fluid and a hypercaloric diet for weight loss, and subcutaneous enrofloxacin (15 mg) for clinical signs of infection. At the study endpoint, or if pre-defined humane endpoints were met (>20% pre-experiment weight loss, severe distress/pain unrelievable by supportive therapy),^16^ rats were euthanized with a sevoflurane and potassium overdose before histologic evaluation. The arrive checklist is provided as supplement.

### Experimental preparation

The experimental preparation and ECMO setup have been described in detail previously.^17,18^ Rats were anaesthetized (sevoflurane 2.5%, buprenorphine 50 mcg/kg BW), intubated and mechanically ventilated (Inspira advanced safety ventilator, Harvard Apparatus). Femoral venous and arterial catheters were surgically inserted for drug administration, haemodynamic monitoring, and blood sampling (baseline, 5 and 15 minutes after ROSC). Three-lead electrocardiogram, end-tidal CO_2_ (etCO_2_), invasive arterial blood pressure (MAP), and pulse oximetry were continuously monitored (IntelliVue MP70 patient monitor, Philips). The ECMO setup (Martin Humbs Engineering) consisted of an open reservoir, draining blood by gravity, roller pump (Masterflex L/S PTFE Tubing Pump, Cole-Parmer), microporous capillary membrane oxygenator, heat exchanger (circulating water bath 1166 D, VWR Polyscience), and silicone tubing. Circuit priming was 15 mL balanced crystalloid solution (Elo-Mel isoton, Fresenius Kabi), heparin (200 IU/kg BW), adrenaline (20 mcg/kg BW) and sodium bicarbonate (1 mmol/kg BW). Sweep gas flow was 400 ml oxygen with 20 ml carbon dioxide admixture. A 14-gauge multistage venous and 22-gauge arterial ECMO cannula were surgically inserted into the right jugular vein and right femoral artery before full heparinization (500 IU/kg BW).

### Experimental procedure

**Figure 1.**
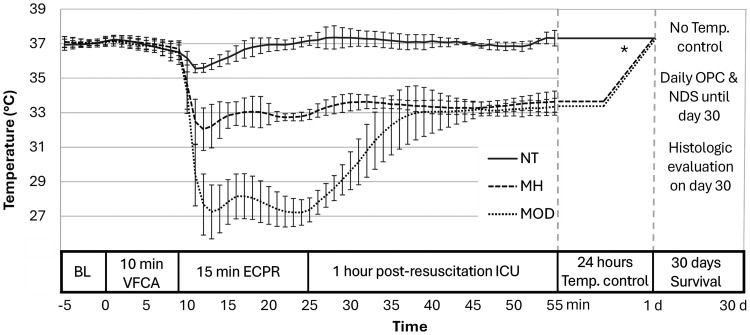
Study Protocol & Temperature profile across study groups. Temperature presented in °C as mean ± standard deviation. Asterix denotes schematic representation of 12 hours maintenance and 12 hours rewarming phases of temperature control. NT: normothermia study group; MH: mild hypothermia study group; MOD: moderate hypothermia study group; BL: baseline; ECPR: extracorporeal cardiopulmonary resuscitation; ICU: intensive care unit; NDS: neurologic deficit score; Temp.: temperature; OPC: overall performance category; VFCA: ventricular fibrillation cardiac arrest.

VFCA was induced with a 2-minute alternating electrical current (50 Hz/5 mA) and confirmed by electrocardiogram and MAP readings, ventilation and anaesthesia were stopped (Figure 1). After 10 minutes no-flow, ECPR started with 100 mL/kg BW ECMO flow rate, adapted to venous return and maintained with fluid boluses, ventilation (20/min, FiO2 1.0) and an additional adrenaline bolus at 2 minutes (10 mcg/kg BW). After 15 minutes ECPR, defibrillation (5 J biphasic) and ECMO weaning were attempted. Ventilation was adapted (65/min, FiO2 0.5), the remaining ECMO circuit blood reinfused, but no vasopressors were given. Sustained ROSC was defined as >20 minutes spontaneous circulation with MAP >50 mmHg. By 30 minutes, all cannulas were removed, the respective vessels ligated, and skin incisions closed. Rats were weaned from ventilator, extubated, and placed in cages with supplemental oxygen.

### Temperature control (study intervention)

Body temperature was continuously monitored with an oesophageal temperature probe (Toes; Mon-a-therm, Medtronic). Before VFCA, Toes was maintained at 37°C using a heated operating table. At the start of ECPR reperfusion, temperature control at the target Toes was immediately initiated at 37°C (NT), 33°C (MH) or 27°C (MOD) with the circulating water bath of the heat exchanger on the ECMO oxygenator set to 42°C, 30°C (maintenance 35°C), or 11°C (maintenance 22°C), respectively. Animals in the MOD group were rapidly rewarmed to 33°C after 15 minutes and weaning from ECMO using the heated operating table. After ROSC and ECMO weaning, temperature control was maintained at 37°C (NT) or 33°C (MH/MOD) for 12 hours before slow rewarming (0.5°C/h) to normothermia (MH/MOD) with the help of a custom heating/cooling ventilator auto-regulated by a telemetric temperature monitoring probe implanted intraperitoneally (Mini-Mitter Co, Sunriver).^17^

### Experimental outcomes

Primary outcome was survival at 30 days with overall performance category (OPC) 1 or 2.

Secondary outcomes included awakening, best neurological function achieved within 30 days and final neurological function at 30 days, cumulative survival, and histopathologic evaluation of the hippocampal CA 1 region of surviving animals.

The rats were assessed daily until 30 days using a 5-point OPC scale (1 = normal, 2 = slight disability, 3 = severe disability, 4 = comatose, 5 = dead)^19^ and a neurologic deficit score (NDS). The NDS consists of 13 tests to evaluate neurological functional deficit on a scale from 0 (normal neurological function) to 100 (braindead): level of consciousness (20 points), respiration (20 points), cranial nerve function (20 points), motor function (10 points), sensory function (10 points), and coordination (20 points).^20^ Good functional outcome was defined as OPC 1 or 2, i.e. animals were able to ambulate, self-care and feed independently, good neurological outcome as NDS ≤10. Awakening was defined as OPC 1 to 3, i.e. animals were attempting to explore spontaneously, ambulating, and reacting to acoustic and sensory stimuli. Best functional and neurological outcome were defined as lowest OPC and NDS ever achieved during the 30-day post resuscitation period.^21^ Final OPC and NDS at 30 days was calculated for surviving animals only.

### Histopathology

Histopathologic evaluation was performed on surviving rats at post-resuscitation week 7–8. Fluid-perfused, formalin-fixed brains were cut into 3 µm coronary sections between bregma 3.5–4.0 to depict the hippocampal cornu ammonis 1 (CA1) region. Sections were stained with haematoxylin and eosin (HE) for descriptive evaluation and vital neuron count, immunohistochemistry was carried out using antibodies against ionized calcium-binding adaptor molecule 1 (Iba1; Wako Chemicals GmbH) and glial fibrillary acidic protein (GFAP, Dako Agilent) to assess microglia and astrocyte activation. Viable neurons with a visible nucleolus were counted at 200-fold magnification in two 250 µm CA1 sectors. Activation of microglial cells (Iba1) and astrocytes (GFAP) was assessed semiquantitatively on a 5-point scale at 100-fold magnification in the entire CA1 region (0 = normal mild staining of physiologic cells, 1 = focal mild increase in signal intensity, 2 = focal moderate increase in signal intensity, 3 = multifocal to diffuse moderate increase in signal intensity, 4 = diffuse marked increase in signal intensity in the entire CA1 region).

### Statistics and data analysis

Discrete data are presented as count (percentage), continuous data as mean (standard deviation). A Chi2 test was used to identify differences between groups in rates of survival, awakening, ROSC, and humane endpoints. One-way ANOVA or Kruskal-Wallis Test, as appropriate, were used to identify differences between groups in baseline, haemodynamic, and blood sampling parameters, as well as OPC, NDS and histologic cell counts. Bonferroni-corrected post hoc analysis was performed to identify differences between individual study groups. A two-sided p < 0.05 was considered statistically significant. Statistical analysis was performed with IBM SPSS version 26.0 for Windows. Sample size was calculated with a power of 80% at a significance level of 0.05 expecting neurologically intact survival of 88% in the MH^17^ and <25% in the NT study group.^18^

## Results

### Baseline characteristics & temperature control

Of 38 rats included in the study, 14 were excluded as per protocol (supplemental Figure 1). Baseline, haemodynamic and resuscitation parameters of the final trial population of 24 rats randomized to the three treatment arms did not differ (Table 1). Cooling was fast and temperature well controlled (Figure 1, Table 1). Blood sampling parameters during the experiment are presented in supplemental Table 1.

**Table 1.** Baseline characteristics.

		Study groups			
	Total	NT 37°C	MH 33°C	MOD 27°C	
Baseline	(n = 24)	(n = 8)	(n = 8)	(n = 8)	p
Preparation					
Weight, grams (SD)	454 (44)	454 (50)	456 (44)	451 (43)	0.966
Anaesthesia before VFCA, min (SD)	118 (22)	131 (25)	113 (17)	109 (21)	0.119
Surgical preparation, min (SD)	64 (15)	67 (15)	62 (18)	62 (20)	0.777
Resuscitation & ICU					
ECMO flow rate, ml/kg/min (SD)	112 (17)	109 (10)	123 (16)	105 (20)	0.095
Spontaneous CV, n (%)	21 (88)	7 (88)	8 (100)	6 (75)	0.319
Time until extubation, min (SD)	42 (15)	39 (17)	46 (14)	40 (18)	0.697
Mean arterial pressure (MAP)					
MAP BL, mmHg (SD)	98 (16)	96 (8)	95 (24)	103 (10)	0.513
MAP VFCA, mmHg (SD)	9 (3)	8 (3)	9 (2)	10 (3)	0.435
MAP ECPR, mmHg (SD)	48 (12)	42 (15)	47 (8)	55 (12)	0.110
MAP ICU, mmHg (SD)	59 (18)	48 (18)	62 (13)	65 (22)	0.194
Temperature (toes)					
Toes BL, °C (SD)	37.0 (0.2)	37.0 (0.2)	37.1 (0.2)	37.0 (0.2)	0.757
Toes VFCA, °C (SD)	36.7 (0.4)	36.8 (0.3)	36.8 (0.4)	36.6 (0.4)	0.441
Toes ECPR, °C (SD)		36.5 (0.2)*^†^	32.7 (0.4)*^‡^	27.6 (0.6)^†‡^	**<0.001**
Toes ICU, °C (SD)		37.1 (0.3)*^†^	33.4 (0.3)*^‡^	31.1 (1.7)^†‡^	**<0.001**
Cooling Rate, °C/min (SD)		0.5 (0.2)*^†^	2.9 (1.2)*^‡^	4.5 (1.3)^†‡^	**<0.001**
Time to Target Temp, min (SD)		0 (0)*^†^	1.3 (0.5)*	1.9 (0.9)^†^	**<0.001**

Data presented as mean ± standard deviation (SD). NT: normothermia study group; MH: mild hypothermia study group; MOD: moderate hypothermia study group. BL: baseline; ECPR: extracorporeal cardiopulmonary resuscitation; ICU: intensive care unit; MAP: mean arterial pressure; ROSC: return of spontaneous circulation; Toes: oesophageal temperature; VFCA: ventricular fibrillation cardiac arrest. Bold typeset denotes statistically significant group differences. Asterix (*), dagger (†) and double dagger (‡) denote difference between respective study groups after Bonferroni correction for multiple comparisons (p < 0.05).

### Primary outcome

Survival to 30 days with OPC 1 or 2 occurred in 2 of 8 rats (25%) in the normothermic, 5 of 8 rats (63%) in the mild hypothermic, and 3 of 8 rats (38%) in the moderate hypothermic study groups and did not statistically differ between groups (Table 2).

**Table 2.** Primary and secondary outcomes.

		Study groups				
	Total	NT 37°C	MH 33°C	MOD 27°C		
Outcome	(n = 24)	(n = 8)	(n = 8)	(n = 8)		P
Primary outcome						
Survival 30 days with OPC ≤2, n (%)	10 (42)	2 (25)	5 (63)	3 (38)		0.301
Secondary outcomes						
Sustained ROSC, n (%)	21 (88)	6 (75)	8 (100)	7 (88)		0.319
Humane endpoint, n (%)	4 (16)	0 (0)	2 (25)	2 (25)		0.301
Survival time, days (IQR)	6 (0–30)	0 (0–23)	30 (5–30)	5 (0–30)		0.175
Neurological outcomes						
Awakening (OPC ≤3), n (%)	15 (63)	2 (25)*	7 (88)*	6 (75)		**0.024**
Best OPC (IQR)	3 (2–5)	5 (4–5)*	2 (1–3)*	3 (2–4)		**0.030**
Best NDS (IQR)	27 (3–100)	100 (52–100)*	3 (2–40)*	20 (5–72)		**0.048**
Final OPC 30-day survivors (IQR)	2 (1–2)	2 (2–2)	1 (1–2)	2 (1–2)		0.368
Final NDS 30-day survivors (IQR)	3 (2–4)	4 (3–4)	2 (1–2)^‡^	5 (3–5)^‡^		**0.044**
Histopathologic outcomes – 30-day survivors	Total	NT 37°C	MH 33°C	MOD 27°C	Naïve	
	(n = 18)	(n = 2)	(n = 5)	(n = 3)	(n = 8)	
Viable Neurons HE (IQR)	60 (57–65)	48 (33–63)	60 (57–63)	55 (51–69)	61 (59–67)	0.680
Iba1 (IQR)	0 (0–0)	2 (0–3)	0 (0–2)	0 (0–2)	0 (0–0)	0.228
GFAP (IQR)	0 (0–1)	2 (1–2)	1 (0–1)	1 (1–1)	0 (0–0)	**0.010**

Data presented as mean ± standard deviation (SD) or median and interquartile range (IQR). NT: normothermia study group; MH: mild hypothermia study group; MOD: moderate hypothermia study group; ROSC: return of spontaneous circulation; HE: haematoxylin and eosin staining; Iba1: ionized calcium-binding adaptor molecule 1; GFAP: glial fibrillary acidic protein; NDS: neurologic deficit score; OPC: overall performance category. Bold typeset denotes statistically significant group differences. Asterix (*) and double dagger (‡) denote difference between respective study groups after Bonferroni correction for multiple comparisons (p < 0.05).

### Secondary outcomes

**Figure 2.**
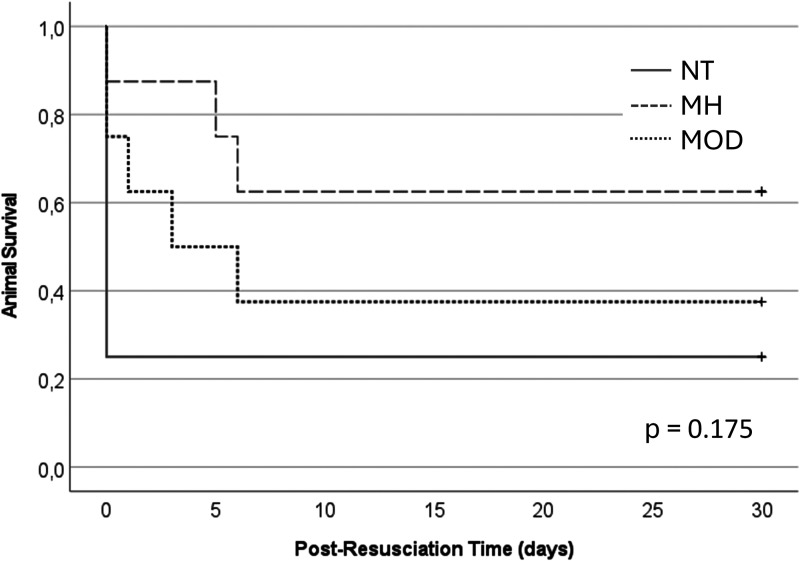
Cumulative survival across study groups. NT: normothermia study group; MH: mild hypothermia study group; MOD: moderate hypothermia study group.

**Figure 3.**
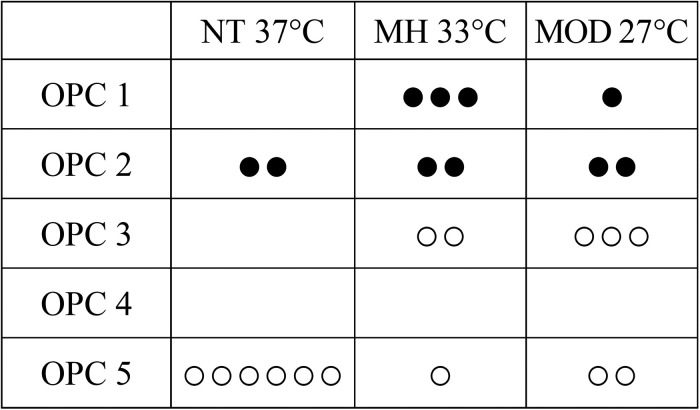
Best OPC across study groups. Each dot represents a rat, ● survived to study endpoint, ○ died before study endpoint, depicting the lowest OPC achieved within the 30-day observation period. NT: normothermia study group; MH: mild hypothermia study group; MOD: moderate hypothermia study group; OPC: overall performance category.

There was no statistically significant difference in cumulative survival between study groups (Figure 2). The best and final OPC for each animal are shown in Figure 3. Rate of awakening, best OPC and best NDS differed statistically significant between groups, the MH group showing the best outcome (Table 2). All 10 rats that survived to study endpoint had good functional and neurological outcome on final examination, the lowest NDS was found in MH rats.

### Humane endpoints & complications

Fourteen rats did not survive to study endpoint. Three rats (2 NT, 1 MOD) did not achieve sustained ROSC. Six rats (4 NT, 1 MH, 1 MOD) never awoke after ROSC. Four rats (2 MH, 2 MOD) were euthanized for humane endpoints on day 3 (MOD; necrotic hind-legs, peritonitis on post-mortem examination), day 6 (MH; weight loss >20%), day 7 (MH; pain/distress), and day 8 (MOD; necrotic hind-legs). One rat (MOD) died on day 3 with post-mortem findings of peritonitis. All other rats had unremarkable post-mortem examinations.

### Histopathologic evaluation

**Figure 4.**
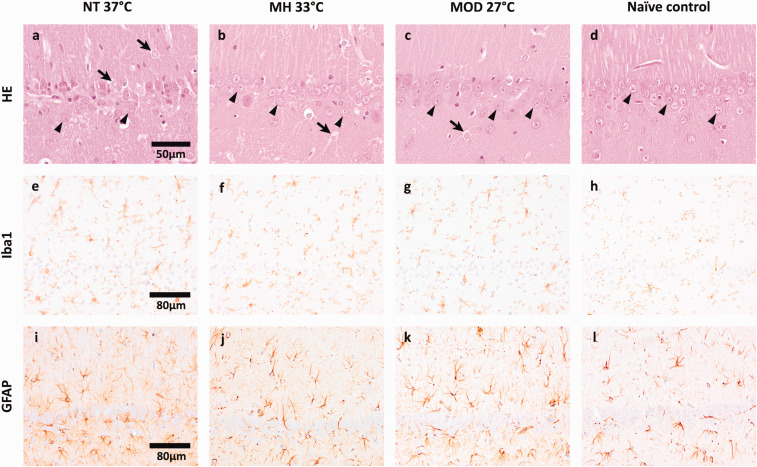
Representative images of the histologic lesions in the CA1 region of CA animals compared to naïve control animals. (a) Reduced number of viable neurons (arrowheads) and multiple activated astrocytes with increased eosinophilic cytoplasm (arrows) in a normothermic CA animal; (b–c) Multiple layers of viable neurons (arrowheads) are present in mild and moderate hypothermic CA animals, but they also show activated astrocytes (arrows); (d) Naïve control animal with multiple layers of viable neurons (arrowheads) in the physiologic hippocampal CA1 region; (a–d) HE-staining. e: Moderate increase of staining intensity in microglia in a normothermic CA animal; (f–g) Mild increase of staining intensity in microglia in mild and moderate hypothermic CA animals; (h) Normal expression of Iba1 in microglia of a control animal; e–h Iba1-IHC. (i) Moderate increase of staining intensity in astrocytes in a normothermic CA animal; (j–k) Mild increase of staining intensity in astrocytes in mild and moderate hypothermic CA animals; (l) Normal expression of GFAP in astrocytes of a control animal; i–l: GFAP-IHC. NT: normothermia study group; MH: mild hypothermia study group; MOD: moderate hypothermia study group; Naïve control: untreated control animals; HE: haematoxylin: and eosin staining; Iba1: immunohistochemistry with primary antibody against ionized calcium-binding adaptor molecule 1; GFAP: immunohistochemistry with primary antibody against glial fibrillary acidic protein.

For histological comparisons, 8 additional naïve rats of similar size and age were used. All rats except for one NT rat showed physiologic structure of the hippocampal CA1 region in HE-staining. Viable neuron counts of the 10 surviving rats did not differ between study groups or when compared to naïve rats (Table 2). One of the 2 NT rats showed loss of viable neurons (33), predominantly in the medial portion of the CA1 region, and activated microglia and astrocytes with gemistocytic appearance in HE-staining. This was confirmed by immunohistochemistry, in which this animal showed increased microglial (grade 3) and astrocyte (grade 2) activity. Two of the 5 MH and 1 of the 3 MOD rats had increased microglial activity (grade 2). Astrocytes were mildly activated (grade 1) in the second NT, 2 MH and all MOD rats, and moderately activated (grade 2) in 1 MH rat. Images of the most affected rats in the respective groups compared to naïve controls in all staining methods are presented in Figure 4.

## Discussion

In this 10-minute VFCA ECPR rat model, no statistically significant difference in survival to 30 days could be demonstrated between cold reperfusion to 33°C or 27°C body temperature for 15 minutes, followed by temperature control at 33°C for 12 hours, or normothermic reperfusion and 12-hour temperature control at 37°C body temperature. However, the 38% absolute difference in survival rate between the MH and NT study group suggests that the study was underpowered to detect this meaningful survival benefit.

## Mortality and short-term outcome

Sustained ROSC was achieved in 88% of rats, comparable to our studies and the literature.^17,18,22,23^ The post-resuscitation mortality rate was higher than expected (37% vs. 12%).^17^ The current study employed a longer 15-minute ECPR duration instead of 2 minutes to achieve cooling and haemodynamic stability in the 27°C study group. Some of the post-resuscitation mortality may be attributable to ECMO-related complications from the increased ECMO runtime, such as air embolism, thromboembolism, or systemic inflammatory response. These limitations of custom-made ECMO setups and post-resuscitation care in small animal models might be overcome by choosing earlier study endpoints. When considering the Kaplan-Meier plots for this study (Figure 2), higher survival rates were observed in the MH and MOD study group during the first post-resuscitation days. On the other hand, long-term survival models are required to assess neuronal regenerative potential^24,25^ or cognitive function.^26^

## Histopathological outcomes and study endpoint

Only 1 NT rat of all 10 surviving rats had substantial histological damage. This is consistent with our previous findings,^17^ whereas significant loss of neurons was observed in a study similarly designed to ours.^13^ The amount of neurological damage after CA has been shown to be dependent on the examination time, possibly through neuronal repopulation.^24^ The mode of CA has been shown to affect low-flow profiles, cardiovascular and neurological damage.^27^ Whether differences in histological damage between the two studies are attributable to the mode of CA (asphyxia or VF), duration of CA (8-minute or 10-minute), the time point of examination (3 days or 7–8 weeks) or the fact that animals with neurological and histological damage might have died and were thus unavailable for histological examination in this study is unknown. Mild to moderate microglial and astrocyte activation was observed in all but 2 MH rats, despite the late timepoint of histologic evaluation and normal viable neuron counts in the respective animals, indicating an inflammatory response still ongoing.

## Neurological outcomes

Interestingly, all secondary neurological outcome parameters, including awakening rate, best overall and neurological function at any time point and neurological function of surviving rats favoured MH reperfusion. Currently, the Core Outcome Set recommends reporting outcome after CA at specific time points only.^28^ The best CPC project highlighted the importance of patients who awaken after CA but later die from intensive care-related complications.^21^ Identifying patients *without* irreversible ischaemia-reperfusion injury may inform preventive and therapeutic strategies and represents a crucial patient-centric outcome and should therefore be reported in all studies, especially when evaluating neuroprotective interventions.

## Temperature control with ECPR

Meta-analyses of retrospective studies mostly support the neurological benefit of temperature control at 33–34°C for 24 hours,^29–32^ as is currently recommended by the Extracorporeal Life Support Organization,^33^ but included studies had small sample sizes and were heterogeneous. To date, there is no randomized controlled clinical trial investigating different temperature control protocols in ECPR patients.^34^

Several randomized controlled ECPR animal studies have investigated different reperfusion temperatures after cardiac arrest, but there is considerable heterogeneity between these studies, conducted over a period of more than 30 years, in terms of cooling rates (225°C/h to 9°C/h), temperature control duration (30 minutes to 20 hours), and study endpoints (3 days to 14 days). Mild hypothermic (32–34°C) reperfusion demonstrated improved neurological outcome compared to normothermic (37–37.5°C) reperfusion in rats, pigs, and dogs.^13,35–38^ One rat study showed no survival benefit but reduced histological damage, which could be attributable to a shorter CA duration.^23^ Deep hypothermic reperfusion temperatures (15–24°C) did not prove beneficial.^35,39^ Moderately hypothermic reperfusion (27–30°C) compared to mild hypothermic reperfusion improved neurological outcome in one study,^13^ was equipotent in two studies,^35,37^ and showed a trend towards reduced survival in this study. The reasons for the variable results of moderately hypothermic ECPR reperfusion remain to be evaluated (mode and duration of CA, time of outcome assessment, temperature level 27°C or 30°C, as discussed above). Of note, animals in the MOD group of this study experienced two rewarming phases, early rapid rewarming from 27°C to 33°C within 15 minutes after ECRP weaning, followed by 12 hours of temperature control at 33°C, before slow rewarming to normothermia (0.5°C/h). Rewarming to 33°C was deemed necessary to ensure rhythmic and hemodynamic stability of the animals after the extracorporeal support and ICU phase. There is low overall certainty on the optimal rewarming rate after induced mild hypothermia following cardiac arrest.^7,9^ The rewarming phase has been associated with increased inflammatory response and brain ischaemia in cardiac arrest patients,^40,41^ and faster rewarming (4°C/h vs. 0.5°C/h) has been associated with reduced survival and neurological function after asphyxia cardiac in rats.^42^ A continuously slow rewarming rate (0.5°C/h) following ECPR reperfusion at 27°C might improve the neuroprotective effect in this study group.

Overall, both animal and clinical studies suggest that mild hypothermic reperfusion induced by ECPR improves neurological outcome and survival rates, whereas deeper levels of hypothermia may worsen outcome. Further animal research is necessary to optimize reperfusion temperature protocols in ECPR and to inform future clinical trials in this area.

## Limitations

This study has several limitations. First, the power calculation for the primary outcome was based on a previous study with 88% good neurological outcome in the treatment group,^17^ but only 63% of animals achieved good neurological outcome in the current study, leaving the current study underpowered to draw definitive conclusions for the primary outcome. Second, blinding was not possible during the study intervention, potentially influencing results. However, post-resuscitation care and outcome evaluation were blinded to minimize bias. Third, the nature of this small animal model precluded prolonged intensive care, which might improve survival. Fourth, the absence of routine periprocedural antibiotics and removal of the peritoneal temperature probe on day 2 might have increased the infectious and thus mortality risk in the post-resuscitation period. Fourth, more detailed cognitive testing, such as the Morris water maze, was not performed as a study endpoint. Implementing such tests might have allowed for quantifying functional differences between study interventions. Last, the study was conducted on young, healthy male rats, limiting direct applicability of results to human patients who are usually older with a cardiovascular medical history.

## Conclusion

In conclusion, ECPR with 33°C reperfusion did not statistically significantly improve survival after prolonged VFCA when compared with 37°C or 27°C reperfusion but was neuroprotective as measured by rate of awakening and best neurological function achieved. ECPR resulted in viable neuron counts comparable to naïve rats.

## Supplemental Material

sj-pdf-1-jcb-10.1177_0271678X241281485 - Supplemental material for Neuroprotection with hypothermic reperfusion and extracorporeal cardiopulmonary resuscitation – A randomized controlled animal trial of prolonged ventricular fibrillation cardiac arrest in rats Supplemental material, sj-pdf-1-jcb-10.1177_0271678X241281485 for Neuroprotection with hypothermic reperfusion and extracorporeal cardiopulmonary resuscitation – A randomized controlled animal trial of prolonged ventricular fibrillation cardiac arrest in rats by Ingrid Magnet, Alexandra-Maria Stommel, Christoph Schriefl, Matthias Mueller, Michael Poppe, Juergen Grafeneder, Christoph Testori, Andreas Janata, Andreas Schober, Daniel Grassmann, Wilhelm Behringer, Wolfgang Weihs, Michael Holzer, Sandra Hoegler and Florian Ettl in Journal of Cerebral Blood Flow & Metabolism
