# Supplementary material for: Neuroprotection with hypothermic reperfusion and extracorporeal cardiopulmonary resuscitation – A randomized controlled animal trial of prolonged ventricular fibrillation cardiac arrest in rats
Source: J Cereb Blood Flow Metab. 2024 Sep 9;45(3):476–85. doi: 10.1177/0271678X241281485 (PMC11574926; doi:10.1177/0271678X241281485)
Supplement: sj-pdf-1-jcb-10.1177_0271678X241281485 - Supplemental material for Neuroprotection with hypothermic reperfusion and extracorporeal cardiopulmonary resuscitation – A randomized controlled animal trial of prolonged ventricular fibrillation cardiac arrest in rats [file sj-pdf-1-jcb-10.1177_0271678X241281485.pdf]

Neuroprotection with Hypothermic Reperfusion and Extracorporeal Cardiopulmonary Resuscitation – A Randomized Controlled Animal Trial of Prolonged Ventricular Fibrillation Cardiac Arrest in Rats

Ingrid Magnet MD[1], Alexandra-Maria Stommel MD[1], Christoph Schriefl MD, PhD[1], Matthias Mueller MD[1], Michael Poppe MD[1], Juergen Grafeneder MD[1], Christoph Testori MD[1], Andreas Janata MD[1], Andreas Schober MD[2], Daniel Grassmann MD[3], Wilhelm Behringer MD[1], Wolfgang Weihs DVM[1], Michael Holzer MD[1], Sandra Hoegler DVM[4], Florian Ettl MD[1]

Author affiliations:

[1] Department of Emergency Medicine, Medical University of Vienna, Waehringer Guertel 18-20, 1090 Vienna, Austria

[2] Department of Cardiology, Klinik Floridsdorf, Bruenner Straße 68, 1210 Vienna, Austria

[3] Emergency Medical Service Vienna, Radetzkystraße 1, 1030 Vienna, Austria

[4] Unit of Laboratory Animal Pathology, University of Veterinary Medicine Vienna, Veterinaerplatz 1, 1210 Vienna, Austria

Corresponding Author: Alexandra-Maria Stommel
Waehringer Guertel 18-20 / 6D, 1090 Vienna
alexandra-maria.stommel@meduniwien.ac.at

Running Title: Neuroprotection with Hypothermic ECPR in Rats

Keywords: extracorporeal cardiopulmonary resuscitation, ventricular fibrillation cardiac arrest, neuronal damage, temperature control, rat model

TABLE OF CONTENTS

Supplemental Figure 1 .... page 3

Supplemental Table 1 .... page 4

SUPPLEMENTAL MATERIAL

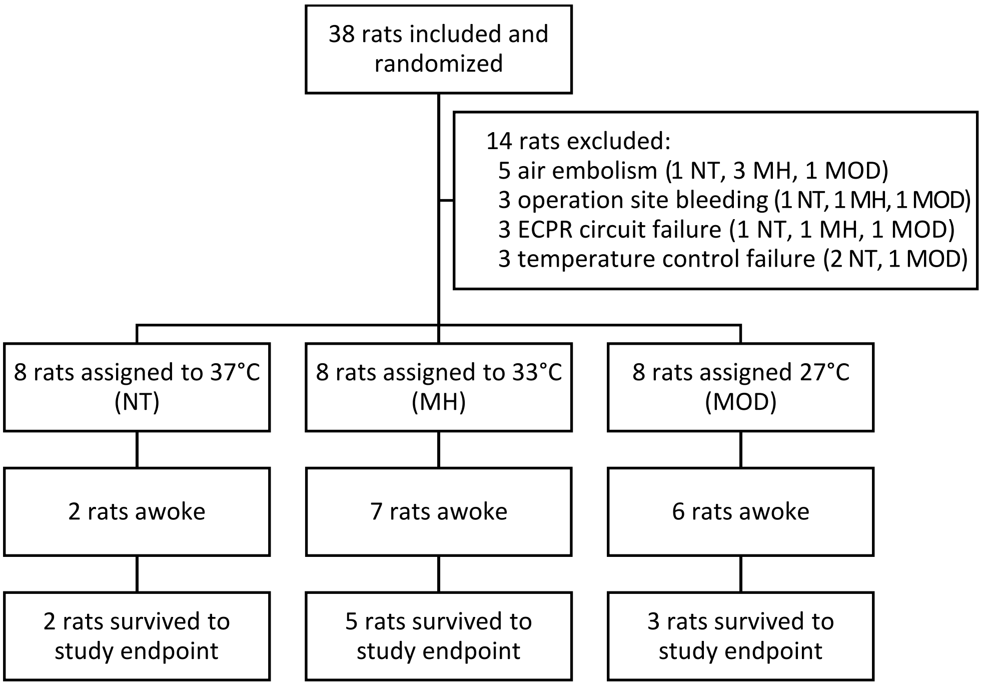

Supplemental Figure 1: Trial profile. ECPR, extracorporeal cardiopulmonary resuscitation. NT, normothermia study group; MH, mild hypothermia study group; MOD, moderate hypothermia study group.

**Blood Gas Analysis**			**STUDY GROUPS**		
	Total (n = 24)	NT 37°C (n = 8)	MH 33°C (n = 8)	MOD 27°C (n = 8)	p
**Blood Gas Analysis at Baseline**					
pH BL (SD)	7.42 (0.04)	7.43 (0.04)	7.43 (0.04)	7.42 (0.04)	0.782
PaO2 BL, mmHg (SD)	149 (58)	141 (51)	131 (40)	174 (75)	0.320
$PaCO_2$ BL, mmHg (SD)	39 (4)	39 (4)	39 (3)	39 (4)	0.990
Hb BL, mg/dl (SD)	14.1 (1.2)	14.1 (0.9)	14.2 (1.4)	14.0 (1.3)	0.941
K^+ BL, mmol/l (SD)	4.1 (0.5)	4.4 (0.5)	4.1 (0.4)	4.0 (0.5)	0.294
Na^+ BL, mmol/l (SD)	138 (3)	137 (3)	139 (2)	139 (3)	0.208
Ca^{2+} BL, mmol/l (SD)	1.19 (0.09)	1.21 (0.08)	1.19 (0.06)	1.18 (0.12)	0.778
BE BL, mmol/l (SD)	1.0 (1.7)	1.2 (1.2)	1.3 (2.1)	0.2 (1.6)	0.375
Glu BL, mg/dl (SD)	173 (30)	177 (28)	170 (15)	170 (43)	0.876
Lac BL, mmol/l (SD)	1.3 (0.4)	1.1 (0.3)	1.4 (0.5)	1.2 (0.3)	0.375
**Blood Gas Analysis at ROSC 5min**					
pH ROSC 5min (SD)	7.13 (0.12)	7.18 (0.15)†	7.17 (0.07)‡	7.01 (0.04)†‡	0.008
PaO2 ROSC 5min, mmHg (SD)	337 (104)	304 (99)	414 (67)‡	281 (99)‡	0.019
$PaCO_2$ ROSC 5min, mmHg (SD)	41 (15)	32 (13)†	35 (7)‡	55 (12)†‡	0.001
Hb ROSC 5min, mg/dl (SD)	9.6 (1.7)	9.3 (2.2)	9.5 (1.5)	10.2 (1.5)	0.613
K^+ ROSC 5min, mmol/l (SD)	3.9 (1.0)	4.7† (1.2)	3.7 (0.5)	3.2† (0.6)	0.010
Na^+ ROSC 5min, mmol/l (SD)	141 (6)	139 (8)	140 (7)	143 (3)	0.475
Ca^{2+} ROSC 5min, mmol/l (SD)	1.19 (0.12)	1.16 (0.12)	1.17 (0.15)	1.23 (0.10	0.521
BE ROSC 5min, mmol/l (SD)	-15.1 (3.2)	-15.5 (3.8)	-14.3 (3.3)	-15.6 (2.9)	0.704
Glu ROSC 5min, mg/dl (SD)	298 (67)	321 (72)	310 (77)	260 (33)	0.191
Lac ROSC 5min, mmol/l (SD)	8.4 (2.0)	9.0 (2.6)	8.2 (1.6)	7.9 (1.8)	0.579
**Blood Gas Analysis at ROSC 15min**					
pH ROSC 15min (SD)	7.18 (0.07)	7.23 (0.05)	7.16 (0.07)	7.16 (0.06)	0.194
PaO2 ROSC 15min, mmHg (SD)	314 (47)	292 (31)	298 (39)	348 (49)	0.081
$PaCO_2$ ROSC 15min, mmHg (SD)	41 (8)	37 (5)	43 (5)	41 (12)	0.477
Hb ROSC 15min, mg/dl (SD)	11.0 (1.0)	10.9 (0.5)	10.9 (1.2)	11.2 (1.2)	0.853
K^+ ROSC 15min, mmol/l (SD)	3.7 (0.6)	4.1 (0.7)	3.4 (0.3)	3.7 (0.6)	0.127
Na^+ ROSC 15min, mmol/l (SD)	140 (3)	139 (4)	141 (2)	141 (2)	0.390
Ca^{2+} ROSC 15min, mmol/l (SD)	1.21 (0.08)	1.15 (0.08)	1.26 (0.08)	1.20 (0.06)	0.091
BE ROSC 15min, mmol/l (SD)	-12.4 (3.4)	-11.2 (5.0)	-12.4 (3.1)	-13.1 (3.0)	0.709
Glu ROSC 15min, mg/dl (SD)	310 (68)	227 (38)*†	358 (55)*	310 (41)†	0.002
Lac ROSC 15min, mmol/l (SD)	7.2 (2.1)	5.9 (2.2)	7.9 (2.3)	7.2 (1.8)	0.347

Supplemental Table 1: Blood gas analysis at Baseline, 5 minutes, and 15 minutes after ROSC. NT, normothermia study group; MH, mild hypothermia study group; MOD, moderate hypothermia study group. BL, baseline; CA, cardiac arrest; ECPR, extracorporeal cardiopulmonary resuscitation; ROSC, return of spontaneous circulation. PaO2, Partial arterial oxygen pressure; PaCO2, Partial arterial carbon dioxide pressure; Hb, haemoglobin; K+, potassium; Na+, sodium; Ca2+, calcium; BE, base excess; Glu, glucose; Lac, lactate. Data presented as mean ±standard deviation (SD). Asterix (*), dagger (†) and double dagger (‡) denote difference between respective study groups after Bonferroni correction for multiple comparisons ($p < 0.05$).
